# Role of Pediatric Otolaryngologist in Pediatric Tracheostomy Code Blue Cases: A New Safety Initiative

**DOI:** 10.1002/lary.70422

**Published:** 2026-02-06

**Authors:** Deepa Shivnani, Miles Jonathan Klimara, M. S. Shruthi, Dnyanesh Balkrishna Amle, Matthew Ern Lin, Ian Kim, R. N. Ashwath Ram, Eswaran Venkat Raman, Gnanam Aram, Mallikarjun Ravi Kobal, Olivia E. Speed, Maie A. St. John, Dinesh Chhetri

**Affiliations:** ^1^ Department of Otorhinolaryngology and Head & Neck Surgery University of Iowa Iowa City Iowa USA; ^2^ Department of Otolaryngology – Head & Neck Surgery University of Iowa Iowa City Iowa USA; ^3^ Department of Otorhinolaryngology and Head & Neck Surgery Mahadevappa Rampure Medical College Kalaburagi Karnataka India; ^4^ Department of Biochemistry All India Institute of Medical Sciences (AIIMS) Nagpur India; ^5^ Department of Head and Neck Surgery David Geffen School of Medicine at University of California, Los Angeles Los Angeles California USA; ^6^ Department of Pediatrics and Department of Disease Prevention Stanford University Palo Alto California USA; ^7^ Department of Pediatric Intensive Care Unit (PICU), Manipal Hospitals Bangalore Karnataka India; ^8^ Department of Pediatric Emergency, Manipal Hospital Bangalore India; ^9^ Department of Pediatrics Mahadevappa Rampure Medical College Kalaburagi Karnataka India; ^10^ Department of Otorhinolaryngology‐ Head & Neck Surgery University of Arkansas for Medical Sciences Little Rock Arkansas USA; ^11^ Department of Head & Neck Surgery UCLA School of Medicine Los Angeles California USA

**Keywords:** multidisciplinary resuscitation team, pediatric otolaryngologist, pediatrics code blue, tracheostomy

## Abstract

**Objective:**

A “Code Blue” is a term to activate an alarm for the resuscitation team for a patient who has a cardiopulmonary arrest. The role of a pediatric otolaryngologist in a tracheostomy‐related code blue case is not clearly defined. We aim to describe the role of pediatric otolaryngologists in pediatric tracheostomy code blue (PTCB) cases.

**Methods:**

This retrospective study analyzed pediatric code blue cases in a tertiary care hospital from January 2019 to December 2022, before and after the implementation of a standardized PTCB that includes a pediatric otolaryngologist in the resuscitation team. Primary outcome variables included response time and survival‐to‐discharge of patients.

**Results:**

The most common reason for code activation was reduced oxygen saturation. The leading cause for the otolaryngology consultation was tube blockage. Tracheostomy tube change was the most common intervention performed. The mean time of otolaryngology arrival was significantly decreased from 14.0 min pre‐implementation to 4.0 min post‐implementation (*p* < 0.001). While including all 48 PTCB events, pediatric otolaryngologist involvement was significantly associated with higher survival‐to‐discharge (94.4% vs. 66.7%, *p* = 0.028). While comparing post‐PTCB protocol implementation versus pre‐implementation, mortality declined from 23.8% to 3.7% with increased discharge rates, although this did not reach statistical significance (*p* = 0.073).

**Conclusion:**

Inclusion of a pediatric otolaryngologist in the resuscitation team reduces time‐to‐arrival of the pediatric otolaryngologist to the code blue activation site. Reduced time to pediatric otolaryngologist arrival and completion of interventions by pediatric otolaryngologist are associated with reduced mortality in PTCB events.

**Level of Evidence:**

3.

## 1 Introduction

Pediatric in‐hospital cardiopulmonary arrest is a relatively rare occurrence. However, with a survival‐to‐discharge rate of less than 50%, any shortcomings in the initial management of a patient's deterioration by healthcare providers (HCPs) may have profound repercussions [1, 2]. Early signs of cardiopulmonary arrest can be preceded by physiological signs of decompensation or deterioration in pediatric patients which offer the opportunity for timely intervention. By identifying and responding to these early indicators, healthcare professionals can play a significant role in preventing cardiopulmonary arrest and improving the outcomes for pediatric patients [3].

While critical airway events are relatively rare, they are common in pediatric academic hospitals, which receive a disproportionate number of patients with complex airway disease, often through large referral networks and specialized programs that offer complex surgical interventions. This setting highlights the need for advanced training and preparation of providers in the care and management of children with critical airway events [4, 5, 6, 7].

Outcome studies of in‐hospital pediatric cardiac arrests demonstrate survival rates of approximately 37%–47% [8, 9, 10]. Compared to adults, children have a higher propensity to suffer resulting physiological decompensation, warranting particular attention to optimization of code activation protocols in this population [4, 11, 12].

Pediatric code blue (PCB) refers to the activation of a specialized resuscitation team aimed at a pediatric patient experiencing cardiopulmonary arrest. Responding to each activation is an assembled team which may include critical care attending physicians, critical care fellows, pediatric residents, respiratory therapists, and nurses who are summoned from the floors, intensive care units, or other hospital departments [13]. Pfeiffer et al. [14] examined team composition, equipment, and training across 18 different sites to compare and contrast code team organization, and identified considerable heterogeneity in the composition of pediatric code teams. This heterogeneity reflects a void in the literature lacking broad systematic descriptions of pediatric code team composition and training.

A major barrier to constructing effective code teams include activation of staff from diverse disciplines and physical locations within the hospital setting. Unlike teams formed within a single unit, such as the pediatric intensive care unit (PICU), where daily collaboration fosters familiarity and teamwork, multidisciplinary teams drawn from different units like the emergency department (ED), PICU, and cardiac intensive care units may lack prior experience working together. More diverse teams are also at higher risk for breakdowns in communication and less effective teamwork during critical events such as code blue activations. Specialized training programs such as simulation exercises can lead to better coordination and improved response to ICU codes. These sessions target diverse groups of HCPs to overcome barriers associated with low familiarity and improve teamwork during emergencies [15, 16, 17]. Surprisingly, there are very few reports that describe programmatic details, such as the team composition, training curricula, and equipment utilized by code teams in pediatric hospital settings [18]. It is difficult to achieve the CPR quality benchmarks as defined by the International Consensus on Cardiopulmonary Resuscitation Guidelines (i.e., chest compression fraction, depth, and rate) even with ongoing in‐hospital monitoring and feedback during in‐hospital cardiac arrests [19, 20]. Though resuscitation teams are routinely assembled within hospitals, the role and influence of the Pediatric Otolaryngologist in tracheostomy‐related code blue situations have not been thoroughly characterized.

Tracheostomies are typically placed to facilitate weaning from mechanical ventilation, maintain a patent airway, or facilitate pulmonary toilet. Although these are valuable clinical interventions, complications can occur in up to 40% of patients, with tube displacement, tube obstruction, pneumothorax, and hemorrhage representing the most common adverse events [21]. Prevention and management of tracheostomy‐related emergencies requires a multidisciplinary approach incorporating standardized protocols, ongoing education and training, appropriate equipment, and patient and carer engagement [22, 23].

To this end, the British National Tracheostomy Safety Project has developed robust protocols to standardize and optimize tracheostomy surveillance and care, which include detailed emergency algorithms and head‐of‐bed signage to optimize tracheostomy‐related emergencies [24]. Similarly, the protocol to manage tracheostomy emergencies developed by Jonathan Graham et al. [25] also promoted the seemingly straightforward measure of calling on the code blue team, but the specific details regarding the involvement or role of otolaryngologists are not provided.

### 1.1 Hypothesis

To date, there has been no literature on the effect of early intervention by pediatric otolaryngologists on pediatric tracheostomy code blue (PTCB) events. As such, the investigators hypothesized a significant benefit in survival with the early involvement of pediatric otolaryngologists in tracheostomized cases.

### 1.2 Objectives

- To compare the survival of pediatric patients with tracheostomy in PCB events before and after the integration of pediatric otolaryngologists into a PTCB team
- To compare the overall survival and time of arrival of otolaryngologists in PCB events before and after the integration of pediatric otolaryngologists into a PTCB team.

## 2 Material and Methods

### 2.1 Study Design

The authors used a retrospective chart review method to collect data on all the pediatric code blue events that occurred between January 2019 and December 2022, with a specific focus on the cohort of tracheostomized children. During this time period, a dedicated PTCB protocol was developed and implemented which included pediatric otolaryngology in the PTCB response team. To ensure uniformity, only PCB activations in tracheostomized patients where a pediatric otolaryngologist was involved were analyzed in both cohorts.

### 2.2 Study Duration

Medical charts from all pediatric patients who experienced a code blue event involving a pediatric otolaryngologist at a single, standalone tertiary care hospital between January 1, 2019, and December 31, 2022, were systematically reviewed and analyzed. These events were stratified based on time relative to the implementation of the PTCB protocol.

### 2.3 Study Population

The study population included all subjects who experienced code blue events during the study period.

Group 1, representing the pre‐implementation phase, covered incidents from January 2019 to December 2020. During this period, standard code blue procedures were followed without the specialized PTCB protocol.

Group 2 is the post‐implementation group, in which incidents from January 2021 to December 2022 (after the implementation of the PTCB protocol from January 1, 2021) were included. Details of all pediatric code blue events were recorded in an electronic database, which was subsequently integrated into the patient's electronic medical records.

### 2.4 Inclusion and Exclusion Criteria

All code blue events which engaged a pediatric otolaryngology specialist for tracheostomized pediatric patients on hospital floors, the ED, pediatric intensive care unit (PICU), and hallways were included for detailed analysis. We excluded all calls involving patients more than 18 years of age and those originating from outpatient clinics, operating rooms, neonatal intensive care units (NICU), or cardiothoracic intensive care units (CTICU) where CPR providers were always available.

### 2.5 Variables and Outcome Measures

- Key variables analyzed included:
- Demographic characteristics: Age, gender, and relevant history of airway surgeries.
- Indications for code blue activation: Specific reasons prompting the code blue call.
- Factors necessitating pediatric otolaryngology consultation: Criteria that led to the involvement of a pediatric otolaryngologist.
- Response times: Time taken for the pediatric otolaryngology specialist to arrive on the scene.
- Therapeutic measures: Specific interventions performed by the pediatric otolaryngologist.
- Clinical outcomes: Final patient outcomes, categorized as either survived to discharge or deceased.

Procedural details such as time to tracheostomy change or airway maneuvers were not uniformly available in retrospective documentation and were not analyzed as they fell outside the primary objectives of this study.

### 2.6 Statistical Analysis

Descriptive and comparative statistics were computed with STATA SE Version 19.0 (StataCorp, College Station, TX, USA). The normality of the data was evaluated using visual inspection of histograms and QQ plots. Mann–Whitney *U* tests were used to compare the means of continuous data. The overall significance of observed changes was analyzed. For frequency distribution comparisons, the differences were evaluated using Fisher's exact test. A *p* value of less than 0.05 was deemed statistically significant.

### 2.7 Pediatric Code Blue Teams

At our institution, a single PCB team responds to all in‐hospital clinical emergencies. There is no separate rapid response team. The institutional code blue team consists of a multidisciplinary group including a pediatric intensive care physician or fellow, a senior pediatric resident, an anesthesiology staff physician or resident, two critical‐care trained nurses, a scribe nurse, an assigned nurse, a security officer, and a dedicated lift operator for staff mobilization. Members of PCB teams participate in a mock code blue simulation program to improve preparedness through monthly drills and mock codes.

The PTCB team was developed to quickly respond specifically to airway emergencies, emphasizing urgent intervention to minimize delays and reduce the need for repeated attempts at airway interventions by primary providers. In addition to the staff included in routine PCB activations, a PTCB activation includes a pediatric otolaryngologist. This composition aims to ensure comprehensive expertise in efficient management of complex airway situations and the hospital's readiness to address critical incidents effectively. Our institution additionally provides structured tracheostomy care education for pediatric residents, nurses, respiratory therapists, and ICU staff. This includes annual simulation training, airway safety drills, and bedside procedural refreshers. First responders receive targeted education on emergency tracheostomy troubleshooting and escalation of protocols.

### 2.8 The PTCB Activation Protocol

The PTCB was officially implemented from January 1, 2021. During the pediatric code blue situation, any member of the health care staff present near the patient must immediately begin life‐saving measures. The PTCB is called by dialing a special extension and saying “Pediatric Tracheostomy Code Blue,” and the floor and room number. This information runs as an indoor announcement over the hospital intercom with the details of the location and specific room number of the patient. Subsequently, separate phone calls are made to the duty mobile numbers of the code blue team personnel to reach the event site.

The pediatric otolaryngology team includes in‐house fellows during the day and on‐call fellows after hours. Trainees are typically first responders. Code timing (daytime vs. overnight/weekends) was not analyzed for response time variability as this was not included in the research objectives.

The goal of the PTCB is to have both personnel and equipment at the bedside within 5 min of the activation call. Activation criteria for the PTCB encompass the following clinical scenarios:

- Severe respiratory distress persists despite appropriate tracheostomy tube suctioning in a tracheostomized child.
- Discovery of an unresponsive state in a tracheostomized child.
- Active seizures were observed in a tracheostomized child.
- Severe respiratory distress, oxygen saturation (SPO2) below 94% even after supplemental oxygen greater than 10 L/min.
- Bradypnea (respiratory rate < 10 breaths/min), gasping, or apnea detected in a tracheostomized child.
- Bradycardia (heart rate < 80 beats per minute) points to clinical deterioration in a child on tracheostomy.

## 3 Results

During the study period, there were 129 PCB activations. Of the 129 PCB activations between January 2019 and December 2022, 48 involved tracheostomized patients and were attended by a pediatric otolaryngologist. These 48 cases were included in the analysis. Among these, 21 occurred before and 27 after implementation of the PTCB protocol.

Considering all otolaryngology‐related PCB cases, 34 (70.8%) of all patients were male, while 14 (29.2%) were female. The majority of cases (70.8%) occurred in children aged 1–5 years, followed by 18.8% in children aged 6–10 years. The mean age of the children was 5.2 years, with a standard deviation of 3.0 years (5.2 ± 3.0). Table S1 details the features of the study subjects before implementation and after implementation of the PTCB protocol.

The most frequent reasons for code activation were low oxygen saturation (37.5%) and tachypnoea (35.4%). Other observed causes included seizures, bradycardia, bleeding through the stoma, bradypnea, and in one instance, a code blue was activated due to staff concern (Table 1). The most frequent indications for consultation with a pediatric otolaryngologist including the pre and post‐implementation group were tracheostomy tube blockage (54%) followed by tracheostomy tube dislodgement (23%). (Table S1).

**TABLE 1:** The year‐wise distribution for code activation where pediatric otolaryngologist was involved.

Reason	Year, *n* (%)				
	2019	2020	2021	2022	Total
Decreased oxygen saturation	4 (44.44)	3 (25.00)	4 (36.36)	7 (43.75)	18 (37.50)
Tachypnea	3 (33.33)	4 (33.33)	4 (36.36)	6 (37.50)	17 (35.42)
Seizure	2 (22.22)	1 (8.33)	1 (9.09)	2 (12.50)	6 (12.50)
Bradycardia	0 (0.00)	2 (16.67)	1 (9.09)	1 (6.25)	4 (8.33)
Bradypnea	0 (0.00)	0 (0.00)	1 (9.09)	0 (0.00)	1 (2.08)
Staff concern	0 (0.00)	1 (8.33)	0 (0.00)	0 (0.00)	1 (2.08)
Stomal bleeding	0 (0.00)	1 (8.33)	0 (0.00)	0 (0.00)	1 (2.08)
Total	9 (18.75)	12 (25.00)	11 (22.92)	16 (33.33)	48 (100.00)

To directly assess the effect of pediatric otolaryngologist involvement on code activation outcomes, the association between intervention by a pediatric otolaryngologist on survival to discharge was calculated using Fisher's exact test, irrespective of whether the event occurred pre‐ or post‐implementation of the PTCB team. The rate of survival to discharge was significantly higher in subjects who received intervention from a pediatric otolaryngologist (94.4%) compared to those who did not receive such intervention (66.7%) (*p* = 0.028) (Table 2).

**TABLE 2:** The outcome of pediatric otolaryngologist involvement in all the tracheostomy code blue cases.

Intervention by pediatric, otolaryngologist *N* (%)	Outcome		*p*
	Deceased	Discharged	
Yes	2 (5.56)	34 (94.44)	0.028
No	4 (33.33)	8 (66.67)	

Similarly, the influence of PTCB implementation with addition of the pediatric otolaryngologist to the code team was explored using the two tailed Fisher exact test. The post‐implementation survival rate (96.3%) trended towards improvement relative to the pre‐implementation survival (76.9%), though this failed to reach statistical significance. (*p* = 0.073) (Table 3).

**TABLE 3:** The outcome of pediatric otolaryngologist involvement in pediatric tracheostomy code blue (PTCB) cases.

Outcome, *N* (%)	Implementation of PTCB		*p*
	Pre‐implementation	Post‐implementation	
Deceased	5 (23.81)	1 (3.70)	0.073
Discharged	16 (76.19)	26 (96.29)	

Time of arrival of the pediatric otolaryngologist to tracheostomy code events was substantially reduced by PTCB team implementation, from 14 min in the pre‐implementation group to 4 min post‐implementation (*p* < 0.0001, Table 4).

**TABLE 4:** Comparison of pediatric otolaryngologist arrival time before and after implementation of PTCB protocol.

PTCB protocol	Median time (min)	IQR	*p*
Pre‐implementation	14.0	5.00	< 0.001
Post‐implementation	4.0	2.00	

Additionally, the association between pediatric otolaryngologists' arrival times and patient survival was analyzed. The timing of pediatric otolaryngologist arrival to the code event was significantly longer in the non‐survivor group (18.5 min) than in patients who survived to discharge (5.0 min, *p* < 0.0001, Table 5). Patients with a history of prior airway surgery had a tendency towards lower mortality than patients who did not, though this did not reach statistical significance (*p* = 0.09, Table 6).

**TABLE 5:** Comparison of pediatric otolaryngologist arrival time between groups with different outcomes.

Patient outcome	Median time (min)	IQR	*p*
Deceased	18.5	2.00	< 0.001
Discharged	5.0	8.00	

**TABLE 6:** Outcome analysis with history of previous airway procedures.

History of airway surgery, *N* (%)	Implementation of PTCB		*p*
	Deceased	Discharged	
Yes	1 (16.67)	24 (57.14)	0.091
No	5 (83.33)	18 (42.86)	

## 4 Discussion

This study followed a group of 48 patients with documented tracheostomy code blue events. Our study found that the majority of cases were between 1 and 5 years of age. In a recent nationwide cohort survey of 15,348 patients, the mean age was 4.5 years for ED cases and 6.2 years for in‐patient cardiac arrest cases [26].

### 4.1 Factors Underlying Code Activations

There were no statistical differences identified regarding the place of code activation before or after the PTCB protocol implementation or between the survived and deceased groups in our study, pointing to an overall similar profile of patients for whom codes were called before and after implementation. Previous studies also likewise have not identified differences in outcomes between cardiac arrest in the intensive care unit (ICU) and cardiac arrest in the general wards [27, 28].

The most frequent reason for code activation through the need for emergency resuscitation in both cohorts was low oxygen saturation (37.5%) followed by tachypnoea (35.4%). Previous work has suggested different warning signs to alert code blue teams before impeding cardiac arrest in pediatric patients [29, 30]. These data, unsurprisingly, diverge somewhat from other studies of cardiopulmonary code activations; for instance, a recent 20‐year retrospective analysis by Acworth et al. [31] found the most common triggers were seizure and respiratory compromise, which accounted for overall 49% of activations. In our study—which examined only patients for whom otolaryngology was consulted and excluded non‐tracheostomized patients from analysis—nearly all codes were respiratory‐related, reflecting the unique challenges faced in the care of tracheostomized patients.

Our statistical evaluation showed that the history of airway surgery is not a critical factor affecting patients' outcomes (*p* > 0.05) and we could not establish a clear association between a patient's history of previous airway procedures with the outcomes. According to published literature, if the child required CPR, this was identified as a significant predictor of the patient's survival. However, mortality related to CPR widely varied according to the inclusion criteria of studies and the study population: Africa (78.1%), Brazil (85.3%), and Spain (66.7%) [32, 33, 34].

### 4.2 Role of Pediatric Otolaryngologist in Code Team

Despite the widespread use of formal rapid response and code blue teams, there remains significant variability in activation protocols, team composition, timeliness of response, and follow‐up. To date, the ideal structure of the pediatric code blue team and its composition have not been established [14, 35]. Successful resuscitation requires specialized skills and teams for advanced airway management [36, 37]. A report from the National Registry of Cardiopulmonary Resuscitation Events suggested that the presence of pediatric residents and fellows improves 24‐h survival for pediatric in‐hospital CPR [38].

Surprisingly, there is very limited data on the otolaryngologist's involvement in the pediatric code blue team or resuscitation team. However, the role of otolaryngologists in complex and emergent surgical airway is well documented [7, 39, 40, 41, 42] Pediatric code blue events in tracheostomized children are frequently airway emergencies which may lead to significant morbidity and mortality if not managed appropriately [12, 43]. We hypothesized, therefore, that a pediatric code blue team with the involvement of a pediatric otolaryngologist would improve the outcomes and survival of tracheostomized children. This concept led to the implementation of the PTCB team, which includes pediatric otolaryngologists as a routine member of code blue responses in tracheostomized children with evidence of impending respiratory distress that may be related to tracheostomy dysfunction.

As compared to adults, children have increased oxygen demands and a low tolerance for apneic events, necessitating rapid engagement of appropriate airway specialists [44, 45]. The arrival time of the pediatric otolaryngologist at the code activation site was decreased from 14 min pre‐implementation to 4 min after PTCB implementation. Ensuring pediatric otolaryngology involvement at the time of code activation facilitates rapid engagement of the pediatric otolaryngologist to pursue emergent interventions. Prior authors have reported arrival times of Pediatric Code Blue teams typically ranging from 1 to 5 min, concordant with the timing of pediatric otolaryngologist arrival time in this study [46, 47, 48, 49, 50, 51, 52]. Accordingly, a non‐significant reduction in patient mortality was observed following the implementation of the PTCB protocol, from 23.8% to 3.7% of codes involving tracheostomized patients with pediatric otolaryngologist involvement, though this did not reach statistical significance (*p* = 0.073). The significant difference between pediatric otolaryngologist arrival between survivor and non‐survivor groups (mean 18.5 min vs. 5 min, *p* < 0.0001) supports the notion that immediate engagement of the pediatric otolaryngologist may confer a protective effect during code blue events for tracheostomized patients.

These data support the hypothesis that timely interventions by pediatric otolaryngologists can improve outcomes in patients with tracheostomy. However, the development of such a team may not be feasible in non‐academic or smaller institutions with combined adult practice. In such resource‐limited environments, the preferred solution would be general otolaryngologists who are trained to contribute to the pediatric code team in difficult airway situations.

### 4.3 Limitations

The major limitation of this study is its retrospective nature with examination pre‐ and post‐implementation of a quality improvement initiative. As with all pre‐post intervention studies, there is a risk of temporal confounders such as staffing changes or evolving institutional practices, which may have influenced outcomes independent of the PTCB protocol. The population is small, limiting the number of mortality events and statistical power. It is unlikely that all confounders have been identified and corrected. Associated comorbidities in tracheostomized children were not described as illness severity at the time of tracheostomy may have influenced outcomes. Similarly, we did not specify prior airway surgical history or specific procedural steps during the code events, as these were inconsistently recorded and not part of our original hypothesis‐driven design.

Another major limitation of our study is that it was conducted in a tertiary care pediatric hospital where a pediatric otolaryngologist is typically on‐site or available within minutes. This level of immediate availability is not replicable in all institutions, thereby affecting the generalizability of our findings. However, this study is the first to investigate the potential role of pediatric otolaryngologists in managing pediatric code blue events in children with a tracheostomy. Future research should seek to address the study's limitations while building from its strengths.

## 5 Conclusion

Inclusion of a pediatric otolaryngologist in the emergency resuscitation team and timely intervention is associated with decreased time‐to‐arrival of pediatric otolaryngology to tracheostomy code blue events. Shorter time from code blue activation to pediatric otolaryngologist arrival may be associated with improved mortality. Future prospective research such as a larger multicenter cohort or longer study duration may further clarify the impact of early involvement of pediatric otolaryngologist on the survival outcomes of tracheostomy emergencies.

## Funding

The authors have nothing to report.

## Ethics Statement

Ethical committee approval for the study has been obtained. This project was conducted with institutional review board approval and was deemed exempt from consent since it was a retrospective study.

## Conflicts of Interest

The authors declare no conflicts of interest.

## Supporting information


**Table S1:** Comparison of characteristics of study subject's pre‐implementation and post‐implementation of PTCB protocol.

## Data Availability

The data that support the findings of this study are available on request from the corresponding author. The data are not publicly available due to privacy or ethical restrictions.

## References

[1] G. Chamberlain , R. Gupta , and A. T. Lobos , “Pediatric Code Blue Event Analysis: Performance of Non‐Acute Health‐Care Providers,” Medical Education Online 27, no. 1 (2022): 2106811, 10.1080/10872981.2022.2106811. 35912470 PMC9347468

[2] M. Bimerew , A. Wondmieneh , G. Gedefaw , T. Gebremeskel , A. Demis , and A. Getie , “Survival of Pediatric Patients After Cardiopulmonary Resuscitation for In‐Hospital Cardiac Arrest: A Systematic Review and Meta‐Analysis,” Italian Journal of Pediatrics 47, no. 1 (2021): 118, 10.1186/s13052-021-01058-9. 34051837 PMC8164331

[3] A. G. Reis , V. Nadkarni , M. B. Perondi , S. Grisi , and R. A. Berg , “A Prospective Investigation Into the Epidemiology of In‐Hospital Pediatric Cardiopulmonary Resuscitation Using the International Utstein Reporting Style,” Pediatrics 109, no. 2 (2002): 200–209, 10.1542/peds.109.2.200. 11826196

[4] A. L. Graciano , R. Tamburro , A. E. Thompson , J. Fiadjoe , V. M. Nadkarni , and A. Nishisaki , “Incidence and Associated Factors of Difficult Tracheal Intubations in Pediatric ICUs: A Report From National Emergency Airway Registry for Children: NEAR4KIDS,” Intensive Care Medicine 40, no. 11 (2014): 1659–1669, 10.1007/s00134-014-3407-4. 25160031

[5] S. M. Nykiel‐Bailey , J. D. McAllister , C. R. Schrock , D. W. Molter , J. K. Marsh , and D. J. Murray , “Difficult Airway Consultation Service for Children: Steps to Implement and Preliminary Results,” Paediatric Anaesthesia 25, no. 4 (2015): 363–371, 10.1111/pan.12625. 25677176

[6] S. S. Siddiqui , S. Janarthanan , M. M. Harish , J. V. Divatia , H. Chaudhari , and R. N. Prabu , “Complications of Tracheal Intubation in Critically Ill Pediatric Cancer Patients,” Indian Journal of Critical Care Medicine 20, no. 7 (2016): 409–411, 10.4103/0972-5229.186222. 27555695 PMC4968063

[7] W. Bai , K. Golmirzaie , C. Burke , et al., “Evaluation of Emergency Pediatric Tracheal Intubation by Pediatric Anesthesiologists on Inpatient Units and the Emergency Department,” Paediatric Anaesthesia 26, no. 4 (2016): 384–391, 10.1111/pan.12839. 26738465

[8] R. S. Phillips , B. Scott , S. J. Carter , et al., “Systematic Review and Meta‐Analysis of Outcomes After Cardiopulmonary Arrest in Childhood,” PLoS One 10, no. 6 (2015): e0130327, 10.1371/journal.pone.0130327. 26107958 PMC4479568

[9] N. Jayaram , J. A. Spertus , V. Nadkarni , et al., “Hospital Variation in Survival After Pediatric In‐Hospital Cardiac Arrest,” Circulation. Cardiovascular Quality and Outcomes 7, no. 4 (2014): 517–523, 10.1161/CIRCOUTCOMES.113.000691. 24939940 PMC4276414

[10] R. Ehrlich , S. M. Emmett , and R. Rodriguez‐Torres , “Pediatric Cardiac Resuscitation Team: A 6 Year Study,” Journal of Pediatrics 84, no. 1 (1974): 152–155, 10.1016/s0022-3476(74)80580-9. 12119940

[11] J. H. Lee , D. A. Turner , P. Kamat , et al., “The Number of Tracheal Intubation Attempts Matters! A Prospective Multi‐Institutional Pediatric Observational Study,” BMC Pediatrics 16 (2016): 58, 10.1186/s12887-016-0593-y. 27130327 PMC4851769

[12] A. Nishisaki , D. A. Turner , C. A. Brown , R. M. Walls , and V. M. Nadkarni , “A National Emergency Airway Registry for Children: Landscape of Tracheal Intubation in 15 PICUs,” Critical Care Medicine 41, no. 3 (2013): 874–885, 10.1097/CCM.0b013e3182746736. 23328260

[13] C. Stewart , J. Shoemaker , R. Keller‐Smith , K. Edmunds , A. Davis , and K. Tegtmeyer , “Code Team Training: Demonstrating Adherence to AHA Guidelines During Pediatric Code Blue Activations,” Pediatric Emergency Care 37, no. 12 (2021): e1658–e1662, 10.1097/PEC.0000000000001307. 29040245

[14] S. Pfeiffer , K. G. Lauridsen , J. Wenger , et al., “Code Team Structure and Training in the Pediatric Resuscitation Quality International Collaborative,” Pediatric Emergency Care 37, no. 8 (2021): e431–e435, 10.1097/PEC.0000000000001748. 31045955 PMC8809371

[15] L. Braun , T. Sawyer , K. Smith , et al., “Retention of Pediatric Resuscitation Performance After a Simulation‐Based Mastery Learning Session: A Multicenter Randomized Trial,” Pediatric Critical Care Medicine 16, no. 2 (2015): 131–138, 10.1097/PCC.0000000000000315. 25647122

[16] A. J. Donoghue , D. R. Durbin , F. M. Nadel , G. R. Stryjewski , S. I. Kost , and V. M. Nadkarni , “Effect of High‐Fidelity Simulation on Pediatric Advanced Life Support Training in Pediatric House Staff: A Randomized Trial,” Pediatric Emergency Care 25, no. 3 (2009): 139–144, 10.1097/PEC.0b013e31819a7f90. 19262421

[17] Y. Lin and A. Cheng , “The Role of Simulation in Teaching Pediatric Resuscitation: Current Perspectives,” Advances in Medical Education and Practice 6 (2015): 239–248, 10.2147/AMEP.S64178. 25878517 PMC4388005

[18] L. J. Knight , J. M. Gabhart , K. S. Earnest , K. M. Leong , A. Anglemyer , and D. Franzon , “Improving Code Team Performance and Survival Outcomes: Implementation of Pediatric Resuscitation Team Training,” Critical Care Medicine 42, no. 2 (2014): 243–251, 10.1097/CCM.0b013e3182a6439d. 24158170

[19] R. M. Sutton , H. Wolfe , A. Nishisaki , et al., “Pushing Harder, Pushing Faster, Minimizing Interruptions… but Falling Short of 2010 Cardiopulmonary Resuscitation Targets During In‐Hospital Pediatric and Adolescent Resuscitation,” Resuscitation 84, no. 12 (2013): 1680–1684, 10.1016/j.resuscitation.2013.07.029. 23954664 PMC3825766

[20] T. M. Olasveengen , A. R. de Caen , M. E. Mancini , et al., “2017 International Consensus on Cardiopulmonary Resuscitation and Emergency Cardiovascular Care Science With Treatment Recommendations Summary,” Circulation 136, no. 23 (2017): e424–e440, 10.1161/CIR.0000000000000541. 29114010

[21] K. A. Wilkinson , H. Freeth , and I. C. Martin , “Are We ‘On the Right Trach?’ the National Confidential Enquiry Into Patient Outcome and Death Examines Tracheostomy Care,” Journal of Laryngology and Otology 129, no. 3 (2015): 212–216, 10.1017/S0022215115000158. 25645673

[22] B. A. McGrath and A. N. Thomas , “Patient Safety Incidents Associated With Tracheostomies Occurring in Hospital Wards: A Review of Reports to the UK National Patient Safety Agency,” Postgraduate Medical Journal 86, no. 1019 (2010): 522–525, 10.1136/pgmj.2009.094706. 20709764

[23] B. A. McGrath , N. Calder , S. Laha , et al., “Reduction in Harm From Tracheostomy‐Related Patient Safety Incidents Following Introduction of the National Tracheostomy Safety Project: Our Experience From Two Hundred and Eighty‐Seven Incidents,” Clinical Otolaryngology 38, no. 6 (2013): 541–545, 10.1111/coa.12177. 24102931

[24] C. Doherty , M. Bowler , S. Monks , et al., “Reduction in Harm From Tracheostomy‐Related Incidents After Implementation of the Paediatric National Tracheostomy Safety Project Resources: A Retrospective Analysis From a Tertiary Paediatric Centre,” Clinical Otolaryngology 43, no. 2 (2018): 674–678, 10.1111/coa.12994. 28977740

[25] J. M. Graham , C. M. Fisher , T. S. Cameron , et al., “Emergency Tracheostomy Management Cognitive Aid,” Anaesthesia and Intensive Care 49, no. 3 (2021): 227–231, 10.1177/0310057X21989722. 33887975 PMC8258718

[26] T. Mir , O. M. Shafi , M. Uddin , M. Nadiger , F. Sibghat Tul Llah , and W. T. Qureshi , “Pediatric Cardiac Arrest Outcomes in the United States: A Nationwide Database Cohort Study,” Cureus 14, no. 7 (2022): e26505, 10.7759/cureus.26505. 35923483 PMC9339595

[27] P. Suominen , K. T. Olkkola , V. Voipio , R. Korpela , R. Palo , and J. Räsänen , “Utstein Style Reporting of In‐Hospital Paediatric Cardiopulmonary Resuscitation,” Resuscitation 45, no. 1 (2000): 17–25, 10.1016/s0300-9572(00)00167-2. 10838235

[28] J. Tibballs and S. Kinney , “A Prospective Study of Outcome of In‐Patient Paediatric Cardiopulmonary Arrest,” Resuscitation 71, no. 3 (2006): 310–318, 10.1016/j.resuscitation.2006.05.009. 17069956

[29] H. Duncan , J. Hutchison , and C. S. Parshuram , “The Pediatric Early Warning System Score: A Severity of Illness Score to Predict Urgent Medical Need in Hospitalized Children,” Journal of Critical Care 21, no. 3 (2006): 271–278, 10.1016/j.jcrc.2006.06.007. 16990097

[30] R. M. Vega , H. Kaur , J. Sasaki , and P. F. Edemekong , “Cardiopulmonary Arrest in Children,” in StatPearls (StatPearls Publishing, 2024). 28613789

[31] J. Acworth , L. Dodson , E. Acworth , and J. McEniery , “Changing Patterns in Paediatric Medical Emergency Team (MET) Activations Over 20 Years in a Single Specialist Paediatric Hospital,” Resusc Plus 3 (2020): 100025, 10.1016/j.resplu.2020.100025. 34223308 PMC8244408

[32] J. Choi , A. Y. Choi , E. Park , S. Moon , M. H. Son , and J. Cho , “Trends in Incidences and Survival Rates in Pediatric In‐Hospital Cardiopulmonary Resuscitation: A Korean Population‐Based Study,” Journal of the American Heart Association 12, no. 3 (2023): e028171, 10.1161/JAHA.122.028171. 36695322 PMC9973657

[33] A. Olotu , M. Ndiritu , M. Ismael , et al., “Characteristics and Outcome of Cardiopulmonary Resuscitation in Hospitalised African Children,” Resuscitation 80, no. 1 (2009): 69–72, 10.1016/j.resuscitation.2008.09.019. 19013705 PMC2706393

[34] J. López‐Herce , C. García , P. Domínguez , et al., “Characteristics and Outcome of Cardiorespiratory Arrest in Children,” Resuscitation 63, no. 3 (2004): 311–320, 10.1016/j.resuscitation.2004.06.008. 15582767

[35] A. I. Sen , R. W. Morgan , and M. C. Morris , “Variability in the Implementation of Rapid Response Teams at Academic American Pediatric Hospitals,” Journal of Pediatrics 163, no. 6 (2013): 1772–1774, 10.1016/j.jpeds.2013.07.018. 23992674

[36] T. Kendirli , A. Caltik , M. Duman , et al., “Effect of Pediatric Advanced Life Support Course on Pediatric Residents' Intubation Success,” Pediatrics International: Official Journal of the Japan Pediatric Society 53, no. 1 (2011): 94–99, 10.1111/j.1442-200X.2010.03128.x. 20337984

[37] A. A. Topjian , R. A. Berg , and V. M. Nadkarni , “Pediatric Cardiopulmonary Resuscitation: Advances in Science, Techniques, and Outcomes,” Pediatrics 122, no. 5 (2008): 1086–1098, 10.1542/peds.2007-3313. 18977991 PMC2680157

[38] A. J. Donoghue , V. M. Nadkarni , M. Elliott , D. Durbin , and American Heart Assocation National Registry of Cardiopulmonary Resuscitation Investigators , “Effect of Hospital Characteristics on Outcomes From Pediatric Cardiopulmonary Resuscitation: A Report From the National Registry of Cardiopulmonary Resuscitation,” Pediatrics 118, no. 3 (2006): 995–1001, 10.1542/peds.2006-0453. 16950990

[39] L. C. Berkow , R. S. Greenberg , K. H. Kan , et al., “Need for Emergency Surgical Airway Reduced by a Comprehensive Difficult Airway Program,” Anesthesia and Analgesia 109, no. 6 (2009): 1860–1869, 10.1213/ane.0b013e3181b2531a. 19713264

[40] N. M. Dalesio , N. Diaz‐Rodriguez , R. Koka , et al., “Development of a Multidisciplinary Pediatric Airway Program: An Institutional Experience,” Hospital Pediatrics 9, no. 6 (2019): 468–475, 10.1542/hpeds.2018-0226. 31088891

[41] A. T. Hillel , V. Pandian , L. J. Mark , et al., “A Novel Role for Otolaryngologists in the Multidisciplinary Difficult Airway Response Team,” Laryngoscope 125, no. 3 (2015): 640–644, 10.1002/lary.24949. 25251732

[42] V. Pandian , T. U. Ghazi , M. Q. He , et al., “Multidisciplinary Difficult Airway Team Characteristics, Airway Securement Success, and Clinical Outcomes: A Systematic Review,” Annals of Otology, Rhinology, and Laryngology 132, no. 8 (2023): 938–954, 10.1177/00034894221123124. 36189709

[43] J. E. Fiadjoe , A. Nishisaki , N. Jagannathan , et al., “Airway Management Complications in Children With Difficult Tracheal Intubation From the Pediatric Difficult Intubation (PeDI) Registry: A Prospective Cohort Analysis,” Lancet Respiratory Medicine 4, no. 1 (2016): 37–48, 10.1016/S2213-2600(15)00508-1. 26705976

[44] J. Fiadjoe and P. Stricker , “Pediatric Difficult Airway Management: Current Devices and Techniques,” Anesthesiology Clinics 27, no. 2 (2009): 185–195, 10.1016/j.anclin.2009.06.002. 19703672

[45] R. Patel , M. Lenczyk , R. S. Hannallah , and W. A. McGill , “Age and the Onset of Desaturation in Apnoeic Children,” Canadian Journal of Anesthesia 41, no. 9 (1994): 771–774, 10.1007/BF03011582. 7954992

[46] S. Bişkin Çetin , M. G. Sezgin , M. Coşkun , F. Sarı , and N. Boztuğ , “Evaluation of Code Blue Notifications and Their Results: A University Hospital Example,” Turkish Journal of Anaesthesiology and Reanimation 51, no. 2 (2023): 105–111, 10.5152/TJAR.2023.22965. 37140575 PMC10210833

[47] E. C. Sterrett , C. M. Myer , J. Oehler , B. Das , and B. T. Kerrey , “Critical Airway Team: A Retrospective Study of an Airway Response System in a Pediatric Hospital,” Otolaryngology–Head and Neck Surgery 157, no. 6 (2017): 1060–1067, 10.1177/0194599817719400. 28849711

[48] C. Welbourn and N. Efstathiou , “How Does the Length of Cardiopulmonary Resuscitation Affect Brain Damage in Patients Surviving Cardiac Arrest? A Systematic Review,” Scandinavian Journal of Trauma, Resuscitation and Emergency Medicine 26, no. 1 (2018): 77, 10.1186/s13049-018-0476-3. 30201018 PMC6131783

[49] K. M. Al‐Aboud and D. M. Al‐Aboud , “Hospital Emergency Codes. An appraisal,” Saudi Medical Journal 31, no. 12 (2010): 1377. 21136006

[50] K. E. Sahin , O. Z. Ozdinc , S. Yoldas , A. Goktay , and S. Dorak , “Code Blue Evaluation in Children's Hospital,” World Journal of Emergency Medicine 7, no. 3 (2016): 208–212, 10.5847/wjem.j.1920-8642.2016.03.008. 27547281 PMC4988111

[51] K. G. Kinney , S. Y. N. Boyd , and D. E. Simpson , “Guidelines for Appropriate In‐Hospital Emergency Team Time Management: The Brooke Army Medical Center Approach,” Resuscitation 60, no. 1 (2004): 33–38, 10.1016/S0300-9572(03)00259-4. 14987781

[52] E. A. Hunt , A. R. Walker , D. H. Shaffner , M. R. Miller , and P. J. Pronovost , “Simulation of In‐Hospital Pediatric Medical Emergencies and Cardiopulmonary Arrests: Highlighting the Importance of the First 5 Minutes,” Pediatrics 121, no. 1 (2008): e34–e43, 10.1542/peds.2007-0029. 18166542

