# Supplementary material for: Role of Pediatric Otolaryngologist in Pediatric Tracheostomy Code Blue Cases: A New Safety Initiative
Source: Laryngoscope. 2026 Feb 6;136(6):2769–76. doi: 10.1002/lary.70422 (PMC13158825; doi:10.1002/lary.70422)
Supplement: Supplementary file 1 — Table S1: Comparison of characteristics of study subject's pre‐implementation and post‐implementation of PTCB protocol. [file LARY-136-2769-s001.docx]

Supplemental Table Ⅰ: Comparison of Characteristics of study subject’s pre-implementation and post-implementation of PTCB protocol

| Characteristic, N (%) | Pre-Implementation | Post-Implementation | p-value |
| --- | --- | --- | --- |
| Age (years), Mean (SD) | 5.14 (3.23) | 5.19 (2.96) | 0.672 |
| Gender |  |  | 0.750 |
| Female | 7 (33.33) | 7 (25.93) |  |
| Male | 14 (66.67) | 20 (74.07) |  |
| Code Blue Location |  |  | 0.723 |
| Emergency Room | 5 (23.81) | 5 (18.52) |  |
| PICU | 6 (28.57) | 5 (18.52) |  |
| Radiology Suit | 0 (0.00) | 1 (3.70) |  |
| Ward | 10 (47.62) | 16 (59.26) |  |
| Airway |  |  | 0.517 |
| Acquired | 5 (23.81) | 11 (40.74) |  |
| Congenital | 8 (38.10) | 8 (29.63) |  |
| None | 8 (38.10) | 8 (29.63) |  |
| History of Other Airway Surgery | 10 (47.62) | 15 (55.56) | 0.771 |
| Reason for Code Activation |  |  | 0.222 |
| Bradypnea | 0 (0.00) | 1 (3.70) |  |
| Bradycardia | 2 (9.52) | 2 (7.41) |  |
| Decreased Oxygen Saturation | 7 (33.33) | 11 (40.74) |  |
| Seizure | 3 (14.29) | 3 (11.11) |  |
| Staff Concern | 1 (4.76) | 0 (0.00) |  |
| Stomal Bleeding | 1 (4.76) | 0 (0.00) |  |
| Tachypnea | 7 (33.33) | 10 (37.04) |  |
| Reason for Pediatric Otolaryngologist Call |  |  | 0.054 |
| Airway Anomaly | 1 (4.76) | 0 (0.00) |  |
| Anticipated Difficult Airway | 1 (4.76) | 0 (0.00) |  |
| Damaged Cuff | 2 (9.52) | 0 (0.00) |  |
| Post Airway Surgery | 2 (9.52) | 0 (0.00) |  |
| Stomal/Airway Bleeding | 2 (9.52) | 3 (11.11) |  |
| Tube Block | 11 (52.38) | 15 (55.56) |  |
| Tube Dislodgement | 2 (9.52) | 9 (33.33) |  |
| Airway Secured By |  |  | 0.542 |
| Anesthesiologist | 2 (9.52) | 2 (7.41) |  |
| Pediatric Otolaryngologist | 14 (66.67) | 22 (81.48) |  |
| Intensivist | 5 (23.81) | 3 (11.11) |  |
| Chest Compressions for CPR | 6 (28.57) | 3 (11.11) | 0.153 |
| Intervention performed |  |  | 0.547 |
| Direct Laryngoscopy | 2 (9.52) | 2 (7.41) |  |
| Flexible Scopy | 3 (14.29) | 6 (22.22) |  |
| Tube Reinsertion | 0 (0.00) | 1 (3.70) |  |
| Tracheostoma Widening | 2 (9.52) | 1 (3.70) |  |
| Tube Change | 8 (38.10) | 14 (51.85) |  |
| None | 6 (28.57) | 3 (11.11) |  |
| Intervention by pediatric otolaryngologist | 14 (66.67) | 22 (81.48) | 0.319 |
| Outcome |  |  | 0.073 |
| Died | 5 (23.81) | 1 (3.70) |  |
| Discharged | 16 (76.19) | 26 (96.29) |  |
